# Three new 14-noreudesmane-type sesquiterpenoids from the roots of *Hippophae rhamnoides*

**DOI:** 10.1007/s13659-025-00581-0

**Published:** 2026-02-04

**Authors:** Fatima Abdurrahman Galadanchi, Polina Lopukhina, Sisi Bai, Guohao Dong, Zhongyu Zhou, Haihui Xie, Xiaoyi Wei

**Affiliations:** 1 Guangdong Provincial Key Laboratory of Applied Botany and Key Laboratory of National Forestry and Grassland Administration On Plant Conservation and Utilization in Southern China, South China Botanical Garden, Chinese Academy of Sciences, Guangzhou, 510650 China; 2 South China National Botanical Garden, Guangzhou, 510650 China; 3 University of Chinese Academy of Sciences, Beijing, 100049 China

**Keywords:** *Hippophae rhamnoides*, 14-noreudesmane sesquiterpenoids, Bioactivity

## Abstract

*Hippophae rhamnoides* L. (Elaeagnaceae), commonly known as sea buckthorn, is a medicinal plant valued for its diverse bioactive constituents and broad therapeutic potential. Phytochemical investigation of its roots led to the isolation of three new 14-noreudesmane-type sesquiterpenoids (**1**–**3**), together with sixteen known compounds (**4**–**19**). Their structures and absolute configurations were determined using comprehensive spectroscopic analyses and quantum chemical computations of electronic circular dichroism (ECD) spectra and ^13^C NMR shifts. Three new sesquiterpenoids (**1**–**3**) were tested for their antioxidant, anti-inflammatory, antibacterial, and *α*-glucosidase inhibitory activities. Unfortunately, none exhibited significant activity under the tested conditions. Among the isolated known compounds, all those displaying *α*-glucosidase inhibitory and antibacterial activities are pentacyclic triterpenoids. Hippophamide (**17**) showed significant DPPH and ABTS radical-scavenging activity. These results enriched the chemical profile of *H. rhamnoides* roots.

**Graphical Abstract:**

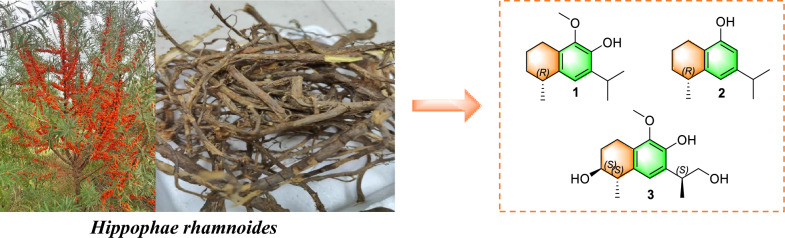

**Supplementary Information:**

The online version contains supplementary material available at 10.1007/s13659-025-00581-0.

## Introduction

*Hippophae rhamnoides* L., commonly known as sea buckthorn, is a member of the Elaeagnaceae family and is native to several regions across Asia and Europe [1, 2]. The plant is predominantly distributed in countries such as China, Turkey, Russia, Mongolia, Tajikistan, India, Greece, Afghanistan, and various other locations [3]. *H. rhamnoides* berries has long been utilized in Chinese traditional medicine to strengthen the spleen, promote digestion, relieve coughs, dispel phlegm, and invigorate blood circulation to dissipate blood stasis [4, 5]. *H. rhamnoides* is not only used in traditional Chinese medicine but also is an actinorhizal plant, which can enter a mutualistic symbiosis with *Frankia*. Extensive phytochemical analyses of *H. rhamnoides* covered various plant organs, such as the leaves [6], fruits [7, 8], branches [9], and seeds [10], and have identified a broad spectrum of secondary metabolites, including terpenoids, alkaloids, volatile oils, flavonoids and steroids. However, chemical studies on the roots remain scarce. So far, only five flavonoids have been documented from the roots [11]. In the ongoing phytochemical research on actinorhizal plant [12, 13], a detailed phytochemical studies of *H. rhamnoides* roots led to the discovery of three new 14-noreudesmane-type sesquiterpenoids (**1**–**3**), along with sixteen known compounds (**4**–**19**). This study presents the processes of extraction and isolation, followed by an in-depth structural analysis of these sesquiterpenoids. Additionally, their biological activities were evaluated, including antioxidant, anti-inflammatory, *α*-glucosidase inhibitory, and antibacterial properties.

## Results and discussion

### Structural elucidation

**Fig. 1:**
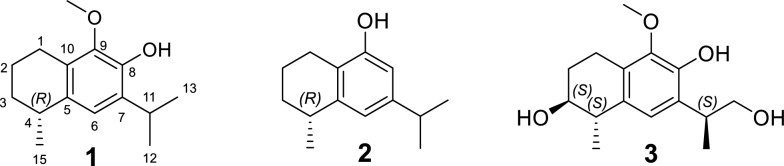
The structures of compounds **1**–**3**

A total of nineteen compounds were successfully isolated and elucidated from the roots of *H. rhamnoides* (Fig. S1, supplementary file), including three newly identified 14-noreudesmane-type sesquiterpenoids (**1**–**3**) (Fig. 1), and sixteen known compounds (**4**–**19**) (Fig. S2, supplementary file).

**Fig. 2:**
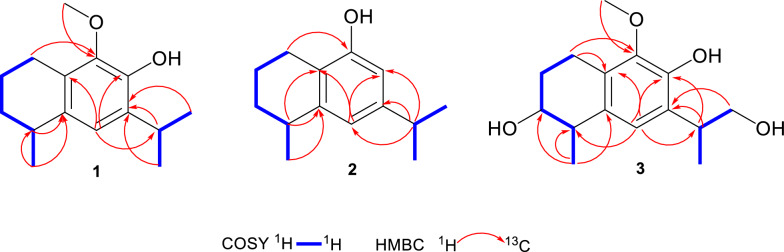
^1^H– ^1^H COSY and key HMBC correlations of **1–3**

Compound** 1** was obtained as a brown oil with [*α*]_D_^25^ = –14.1 (*c* 0.19, MeOH). Its molecular formula was established to be C_15_H_22_O_2_ from its molecular ion peak at *m/z* 234.1614 in the HR-EI-MS spectrum. The ^1^H-NMR spectrum (Table 1) displayed three methyl doublets at δ_H_ 1.27 (3H, d, *J* = 7.0 Hz, H-15), 1.25 (3H, d, *J* = 7.0 Hz, H-12) and 1.24 (3H, d, *J* = 7.0 Hz, H-13), as well as a singlet at δ_H_ 3.78, indicative of an oxygen-substituted methyl group (–OCH_3_). The ^13^C-NMR data (Table 1) in combination with the HSQC spectrum revealed a total of fifteen carbon signals, which were classified as three methyls, one oxygenated methyl, three methylenes, three methines, and five quaternary carbons. The ^1^H-^1^H COSY spectrum of **1** showed two spin-coupled systems of H-1/H-2/H-3/H-4/H-15 and H-12/H-11/H-13 suggesting the presence of a connections of C-1/C-2/C-3/C-4/C-15 and an isopropyl group [14] (Fig. 2). The remaining carbon signals could be ascribed to a benzene ring and an oxygenated methyl. In the HMBC spectrum, δ_H_ 3.25 (H-11), 1.25 (H-12), and 1.24 (H-13) correlated with δ_C_ 132.5 (C-7), and δ_H_ 3.25 (H-11) correlated with δ_C_ 121.2 (C-6) and 143.8 (C-8), suggesting the isopropyl group linked with the benzene ring via C-7. The HMBCs from H-1 to C-5, C-9 and C-10, from H-15 to C-5, from H-6 to C-4, indicated that the moiety of C-1/C-2/C-3/C-4/C-15 were attached to the benzene ring through C-1 linking with C-10, and through C-4 linking with C-5. To meet the requirements of the molecular formula and the presence of five quaternary carbons, a hydroxyl group should be substituted on the benzene ring. The hydroxyl group (δ_H_ 5.60, s) was assigned to C-8 based on its HMBC correlation to C-7. Consequently, the oxygenated methyl group was positioned at C-9. By comparing the measured electronic circular dichroism (ECD) spectra of **1** with the calculated 4*R*-**1** (Fig. 4), the absolute configuration of **1** was determined as 4*R*. Thus, compound **1** was elucidated as (4*R*)-9-methoxy-14-noreudesma-5,7,9-trien-8-ol.

**Table 1:** ^1^H (500 MHz) and ^13^C (125 MHz) NMR data of compounds **1**–**3** in CDCl_3_

	**1**		**2**		**3**	
No.	*δ*_H,_ mult. (*J* in Hz)	*δ*_C_	*δ*_H,_ mult. (*J* in Hz)	*δ*_C_	*δ*_H,_ mult. (*J* in Hz)	*δ*_C_
1	2.71, m	23.9, CH_2_	2.60, m	22.9, CH_2_	2.89, m; 2.75, m	19.9, CH_2_
2	1.70, m; 1.82, m	19.9, CH_2_	1.89, m; 1.76, m	19.5, CH_2_	2.01, dddd (13.7, 8.3, 6.0, 2.7); 1.84, m	27.3, CH_2_
3	1.50, m; 1.89, m	31.5, CH_2_	1.88, m; 1.54, m	30.9, CH_2_	3.81, ddd (7.5, 5.5, 2.5)	72.3, CH
4	2.85, m	32.0, CH	2.89, m	32.6, CH	2.75, m	40.9, CH
5		133.9, C		143.7, C		131.5, C
6	6.84, s	121.2, CH	6.70, d (1.7)	118.7, CH	6.79, s	123.2,CH
7		132.5, C		147.3, C		128.5, C
8		143.8, C	6.52, d (1.7)	109.8, CH		145.0, C
9		143.7, C		153.1, C		144.2, C
10		127.1, C		120.3, C		126.9, C
11	3.25, m	27.4, CH	2.82, m	33.8, CH	3.30, m	36.4, CH
12	1.25, d (7.0)	22.6, CH_3_	1.22, d (7.0)	24.0, CH_3_	1.29, d (7.0)	16.2, CH_3_
13	1.24, d (7.0)	22.6, CH_3_	1.23, d (7.0)	24.1, CH_3_	3.75–3.79, m	68.1, CH_2_
15	1.27, d (7.0)	23.2, CH_3_	1.30, d (7.1)	22.8, CH_3_	1.31, d (7.0)	20.9, CH_3_
OCH_3_	3.78, s	60.2, CH_3_			3.79, s	60.3, CH_3_
Ph-OH	5.60, s		4.65, s		6.08, s

Compound** 2** was obtained as a pale-brown oil with [*α*]_D_^25^ = –20 (*c* 0.11, MeOH). It was found to have the molecular formula C_14_H_20_O according to the molecular ion peak at *m/z* 204.1511 in the HR-EI-MS spectrum. The ^1^H-NMR spectrum (Table 1) displayed three methyl doublets at δ_H_ 1.30 (3H, d, *J* = 7.1 Hz, H-15), 1.23 (3H, d, *J* = 7.0 Hz, H-13), and 1.22 (3H, d, *J* = 7.0 Hz, H-12). The ^13^C-NMR data in combination with the HSQC spectrum revealed a total of fourteen carbon signals, which were classified into three methyls, three methylenes, four methines, and four quaternary carbons (Table 1). Its ^1^H and ^13^C NMR as well as ^1^H-^1^H COSY pattern was closely similar to that of compound **1**, which indicated that compound **2** possessed the 14-noreudesmane skeleton. The key difference between them was the absence of the methoxy group and the presence of an additional aromatic proton signal at δ_H_ 6.52 (1H, d, *J* = 1.7 Hz, H-8) in the ^1^H-NMR spectrum of **2**. The coupling constant between two aromatic protons was 1.7 Hz, which indicated that they are *meta*-position. This is also confirmed by HMBCs from H-11 to C-6, C-7 and C-8. To meet the requirements of the molecular formula and the presence of four quaternary carbons, a hydroxyl group should be substituted on the benzene ring at C-9. The absolute configuration of **2** was further determined as 4*R*-2 by comparing the calculated ECD curve with its experimental values (Fig. 4). As a result, compound **2**, was fully characterized as (4*R*)-14-noreudesma-5,7,9-trien-9-ol.

**Fig. 3:**
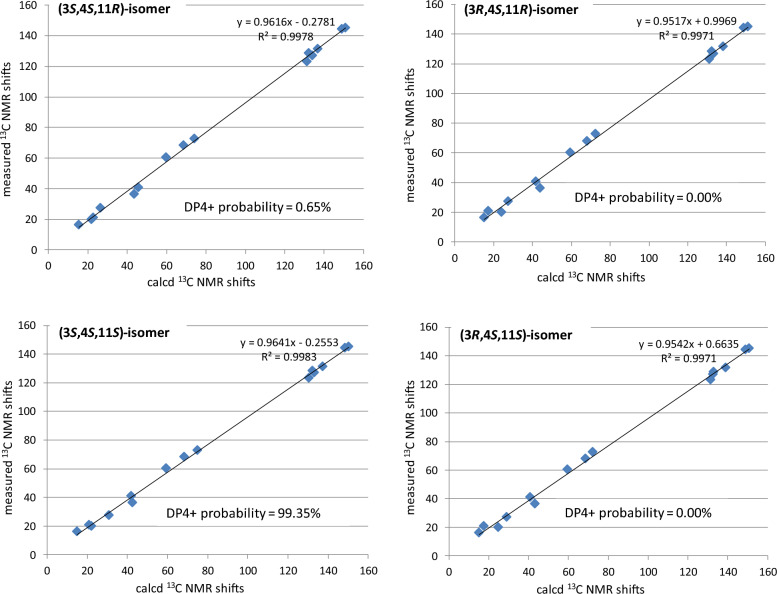
Linear regression analysis of the calculated ^13^C NMR shifts of four possible stereoisomers against the measured shifts of **3** and the goodness of fit test by DP4 + probability analysis

**Fig. 4:**
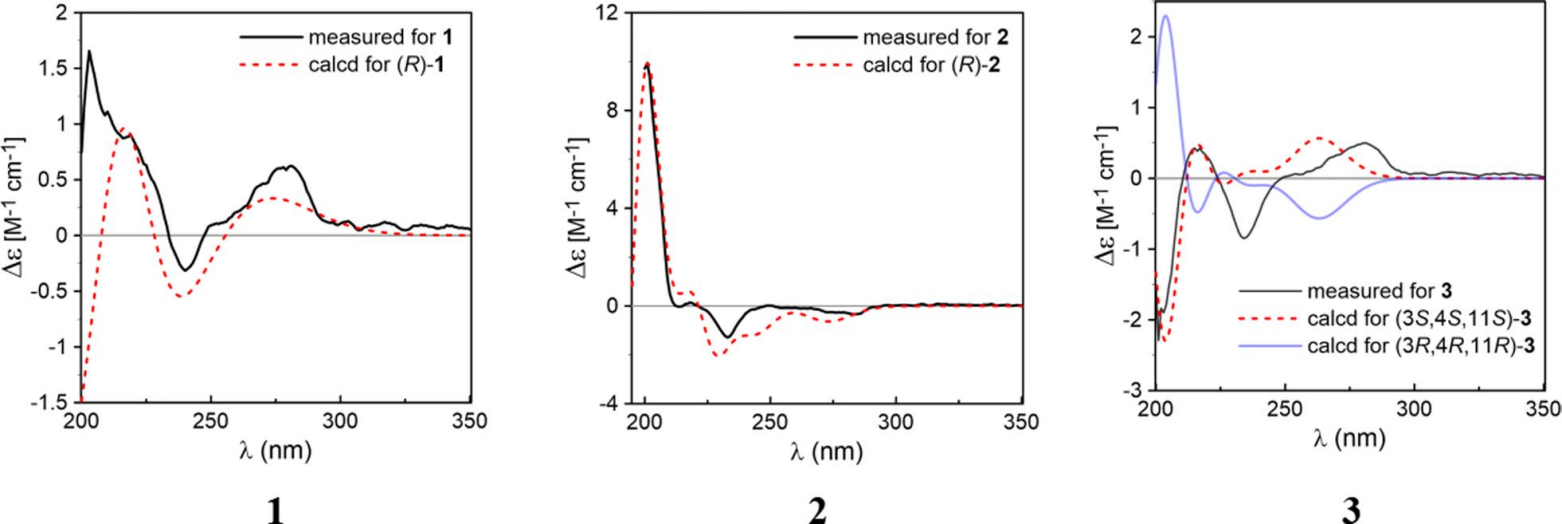
Comparison of the measured and calculated ECD spectra of **1–3** in methanol

Compound** 3** was obtained as a brown oil with [*α*]_D_^25^ = –12.4 (*c* 0.15, MeOH). Its molecular formula was determined as C_15_H_22_O_4_ based on the pseudo-molecular ion at *m/z* 267.1597 [M + H]^+^ observed in positive HR-ESI–MS. The ^1^H-NMR spectrum (Table 1) revealed resonances for one aromatic proton at *δ*_H_ 6.79 (s, H-6), two methyls at *δ*_H_ 1.29 (d, 7.0 Hz, H-12) and 1.31 (d, 7.0 Hz, H-15), and a methoxy group at *δ*_H_ 3.79 (s). The ^13^C-NMR spectrum (Table 1) displayed fifteen carbon signals, five of which are quaternary carbons *δ*_C_ (145.0, 144.2, 131.5, 128.5, and 126.9), four methines, three methylenes, and three methyl carbons. After comparing the ^1^H and ^13^C NMR data with those of compound **1**, it was easy to find** 3** also possesses 14-noreudesmane skeleton. With the help of the HSQC spectrum, all protons were correlated to their respective carbons (Table 1). The ^1^H-^1^H COSY spectrum showed two proton spin-coupled systems of H-1/H-2/H-3/H-4/H-15 and H-12/H-11/H-13 (Fig. 2). C-3 was substituted by a hydroxy group based on the observation of *δ*_H_ 3.80 (m, H-3) and *δ*_C_ (72.3, C-3). C-13 was also linked with a hydroxy group, which was confirmed in the same way like C-3. In the low-field region of ^13^C NMR spectrum, **3** shared highly similar signal pattern of the benzene ring to that of compound **1**. The substitutions of a methoxy group at C-9 and of a hydroxy group at C-8 on the benzene ring were confirmed based on the HMBC observations from H-1 and the methoxy protons to C-9, as well as from H-11 to C-8, respectively. Consequently, compound **3** was identified as 9-methoxy-14-noreudesma-5,7,9-triene-3, 8, 13-triol. In order to assign the relative configuration of **3**, four stereoisomers, (3*S*, 4*S*, 11*R*)-, (3*R*, 4*S*, 11*R*)-, (3*S*, 4*S*, 11*S*)-, and (3*R*, 4*S*, 11*S*)-**3** were subjected to theoretical simulations for ^13^C NMR shifts using the GIAO method [15–18]. The goodness of fit among the predicted ^13^C NMR data of four stereoisomers and the experimental data of **3** were evaluated by the modified DP4 probability (DP4 +) values [19, 20]. As can be seen in Fig. 3, the calculated ^13^C NMR shifts of (3*S*, 4*S*, 11*S*)-isomer showed the best match with the measured data of **3** (DP4 + probability: 99.35%), leading assignment of the relative configurations 3S*, 4S*, 11S*. Then, (3*S*, 4*S*, 11*S*)-**3** was calculated for the ECD spectrum. As can be seen in Fig. 4, the calculated ECD curve was in excellent fit with the experimental spectrum of **3**. Thus, **3** was tentatively assigned to have the absolute configurations 3*S*, 4*S*, 11*S*.

Compounds **1**–**3** were 14-noreudesmane type sesquiterpenoids. Previously, four 14-sesquiterpenoids of this type, namely hipponorterpenes A and B, 6, 9-dihydroxy-1-oxo-14-noreudesma-5,7,9-triene and 6-hydroxy-2-isopropyl-5-methoxy-8-methylnaphthalene-1,4-dione, were also reported from *H. rhamnoides*, but from its berries and juice [14, 21]. Interestingly, such sesquiterpenoids have been isolated from some other natural sources, including *Nicotiana tabacum* (Solanaceae) [22], *Alpinia oxyphylla* (Zingiberaceae) [23], the fungus *Hypoxylon rickii* [24], and the edible mushroom *Flammulina velutipes* [25].

The sixteen known compounds were identified as arjunolic acid (**4**) [26], 18H*α*,3*β*,20*β*-ursanediol (**5**) [27], 3*β*-hydroxyolean-12-en-28-al (**6**) [28], 2*α*,3*β*,19*α*,23-tetrahydroxyolean-12-en-28-oic acid (**7**) [29], erythrodiol (**8**) [30], oleanolic acid (**9**) [31], ursolic acid (**10**) [32], maslinic acid (**11**) [33], ergosterol endoperoxide (**12**) [34], stigmast-4-en-3-one (**13**) [35], *β*-sitosterol (**14**) [36], daucosterol-6'-linoleate (**15**) [37], ( +)-catechin (**16**) [38], hippophamide (**17**) [39], dehydrodiconiferyl alcohol (**18**) [40], 22-*O*-(4-hydroxy-3-methoxy-cinnamy1) docosanoic acid (**19**) [41], (Fig. S2, supplementary file), by comparing their spectroscopic data with previously documented values in literature.

### Antioxidant activity

The antioxidant activity of all compounds was evaluated using two in vitro assays. As summarized in Table 2, compounds **16** and **17** showed notable activity in the DPPH radical scavenging assay, with IC_50_ values of 30.66 μM and 43.15 μM, respectively, compared to *L*-ascorbic acid (IC_50_ = 23.77 μM). In the ABTS cation radical scavenging assay, both compounds **16** and **17** displayed even stronger activity, with IC_50_ values of 13.17 μM and 20.31 μM, respectively, outperforming L-ascorbic acid (IC_50_ = 24.85 μM). Three new compounds **1–3** were tested in the cellular reactive oxygen species (ROS) scavenging assay at 50 μM. All of them showed inhibition rate below 10%. Compared to curcumin (IC_50_ = 16.84 μM), the intracellular antioxidant activity of these compounds was negligible.

**Table 2:** Antioxidant activity of compounds** 1**–**19**

Compounds	DPPH (IC_50_, *μ*M)	ABTS (IC_50_, *μ*M)	Cellular ROS (% inhibition rate)
**1**	> 50	> 50	4.38 ± 2.83^c^
**2**	> 50	> 50	–12.79 ± 4.48^c^
**3**	> 50	> 50	3.17 ± 3.04^c^
**4–15**	> 50	> 50	NT^b^
**16**	30.66 ± 0.64	13.17 ± 0.13	NT^b^
**17**	43.15 ± 0.89	20.31 ± 0.53	NT^b^
**18–19**	> 50	> 50	NT^b^
L-Ascorbic acid^a^	23.77 ± 1.02	24.85 ± 1.17	NT^b^
Curcumin^a^	NT^b^	NT^b^	16.84 ± 0.28 *μ*M

Values are Mean ± SD (*n* = 3)

^a^Positive control

^*b*^*NT* Not tested

^c^Tested at 50 *μ*M

### *α*-Glucosidase inhibitory activity

Compounds **1**–**19** were evaluated for their inhibitory activity against *α*-glucosidase, using corosolic acid as positive control. As shown in Table 3, compound **10** exhibited the most potent inhibitory effect, with an IC_50_ value of 4.81 *μ*M, equivalent to that of corosolic acid (IC_50_ = 5.60 *μ*M). Compounds **4**, **5**, **8**, **9**, and **11** showed moderate activity, with IC_50_ values ranging from 8.96 to 33.58 *μ*M. All active compounds are triterpenoids, while the positive control corosolic acid is also a triterpenoid.

**Table 3:** *α-*Glucosidase inhibitory activity of compounds** 1**–**19**

Compounds	IC_50_ (*μ*M)	Compound	IC_50_ (*μ*M)
**1–3**	> 50	**9**	10.16 ± 0.38
**4**	33.58 ± 1.69	**10**	4.81 ± 0.28
**5**	30.14 ± 2.00	**11**	11.80 ± 1.49
**6–7**	> 50	**12**–**19**	> 50
**8**	8.96 ± 0.40	Corosolic acid^a^	5.60 ± 0.24

Values are Mean ± SD (*n* = 3)

^a^Positive control

### Antibacterial activity

All 19 isolated compounds were evaluated for their antibacterial activity against three bacterial strains: *Staphylococcus aureus* (MSSA, CMCC 26003), methicillin-resistant *Staphylococcus aureus* (MRSA, JCSC 3063), and *Escherichia coli* (EC, ATCC 8739) (Table 4). Minimum inhibitory concentrations (MICs) were determined and compared with positive controls, including kanamycin, vancomycin, and polymyxin. Among the tested compounds, compound **9** exhibited weak antibacterial activity against MSSA and MRSA, with an MIC of 100 *μ*M. Compound **10** displayed weak antibacterial activity against both MSSA and MRSA, with an MIC value of 25 *μ*M. None of the compounds demonstrated antibacterial activity against the Gram-negative strain *E. coli*.

**Table 4:** Antibacterial activity of compounds** 1**–**19** (MIC, *μ*M)

Compounds	MSSA	MRSA	EC
**1–8**	> 100	> 100	> 100
**9**	100	100	> 100
**10**	25	25	> 100
**11–19**	> 100	> 100	> 100
Kanamycin^a^	1.25	> 40	NT^b^
Vancomycin^a^	NT^b^	2.5	NT^b^
Polymyxin^a^	NT^b^	NT^b^	1.25

^a^Positive control

^*b*^*NT* Not tested; (*n* = 3)

### Anti-inflammatory activity

To evaluate the anti-inflammatory potential of three new compounds (**1–3**), their ability to suppress nitric oxide (NO) production was tested using LPS-stimulated RAW 264.7 murine macrophage cells. Compounds **1–3** were tested at concentrations of 50, 25 and 10 *μ*M. However, these compounds exhibited NO production inhibitory rates below 10% at all concentrations tested, compared to the positive control, indomethacin (IC_50_ = 36.29 *µ*M).

## Experimental procedures

### General

Column chromatography (CC) was performed using silica gel (80–100 mesh and 200–300 mesh; Yantai Jiangyou Silica Gel Development Co., Ltd., Yantai, China), Sephadex LH-20 (Pharmacia Fine Chemicals Co., Ltd., Uppsala, Sweden), and reversed-phase C_18_ (YMC Co., Ltd., Japan) materials. Preparative high-performance liquid chromatography (HPLC) was carried out with an LC-6AD pump system equipped with an SPD UV/Vis detector (Shimadzu, Kyoto, Japan), using a Cosmosil column (250 mm × 10 mm i.d., 5 *μ*m particle size, Kyoto, Japan) at a flow rate of 2 mL/min. Thin-layer chromatography (TLC) was performed on precoated silica gel plates (HSGF254; Yantai Jiangyou Silica Gel Development Co., Ltd., Yantai, China), with spot detection achieved by spraying with a 5% vanillin in sulfuric acid solution followed by heating. Proton (^1^H) and carbon (^13^C) nuclear magnetic resonance (NMR) spectra were recorded on a Bruker DRX-500 NMR spectrometer (Bruker Biospin GmbH, Rheinstetten, Germany), with chemical shifts referenced to residual solvent peaks. The spectrometer operated at 500 MHz for ^1^H NMR and 125 MHz for ^13^C NMR. High-resolution electron impact mass spectrometry (HR-EI-MS) and Electrospray ionization mass spectrometry (ESI–MS) data were obtained using a Thermo Scientific DFS magnetic mass spectrometer (Thermo Corporation). Optical rotation values (α_D_) were measured on a Perkin-Elmer 343 polarimeter using methanol (MeOH) as the solvent. Circular Dichroism (CD) spectrum was recorded on a Chirascan circular dichroism spectrometer (Applied Photophysics Ltd., Surrey, UK).

### Plant material

Roots of *Hippophae rhamnoides* were collected in April 2023 from Diqing, Yunnan province, China, by Kunming Zhifen Biotechnology Company, and were botanically authenticated by this company. A voucher specimen (No. Zzy20230414) was deposited at the phytochemistry laboratory, South China Botanical Garden, Chinese Academy of Sciences.

### Extraction and isolation

The air-dried roots were pulverized into a fine powder (14 kg) and extracted three times with 95% aqueous ethanol. The combined filtrates were concentrated under reduced pressure to obtain a crude extract weighing 1007.6 g. The extract was successively partitioned with petroleum ether (4.5 L × 3), ethyl acetate (4.5 L × 3), and *n*-butanol (4.5 L × 3). The resulting fractions were concentrated to dryness under vacuum yielding petroleum ether-soluble (38.0 g), ethyl acetate-soluble (75.2 g), and *n*-butanol-soluble (418.8 g) fractions. The ethyl acetate fraction (75.2 g) underwent purification through silica gel column chromatography (CC), Sephadex LH-20 CC, preparative high-performance liquid chromatography (HPLC), and reversed-phase C_18_ (RP-C_18_) CC to isolate individual compounds. A comprehensive flowchart detailing the phytochemical extraction and isolation procedure for compounds **1–19** is provided in Fig. S2, supplementary file.

### Spectroscopic data new compounds

#### (4*R*)-9-Methoxy-14-noreudesma-5,7,9-trien-8-ol (1)

Brown oil; $[\rm\alpha]^{25}_\mathrm{D}$ = –14.1 (*c* 0.19, MeOH); UV (MeOH) λ_max_ nm (log *ε*): 202 (3.60), 280 (2.44); HR-EI-MS *m/z* 234.1614 (calculated for C_15_H_22_O_2_, 234.1614); ^1^H (500 MHz) and ^13^C (125 MHz) NMR data, see Table 1.

#### (4*R*)-14-Noreudesma-5,7,9-trien-9-ol (2)

Pale brown oil; $[\rm\alpha]^{25}_\mathrm{D}$ = –20 (*c* 0.11, MeOH); UV (MeOH) λ_max_ nm (log *ε*): 203 (3.63), 281 (2.35); HR-EI-MS *m/z* 204.1511 (calculated for C_14_H_20_O, 204.1509); ^1^H (500 MHz) and ^13^C (125 MHz) NMR data, see Table 1.

#### 9-Methoxy-14-noreudesma-5,7,9-triene-3,8,13-triol (3)

Brown oil; $[\rm\alpha]^{25}_\mathrm{D}$ = –12.4 (*c* 0.15, MeOH); UV (MeOH) λ_max_ nm (log *ε*): 203 (3.61), 281 (2.43); HR-ESI–MS (positive) *m/z* 267.1597 (calculated for C_15_H_23_O_4_, 267.1591); ESI–MS (positive) *m/z* 267 [M + H]^+^ and 289 [M + Na]^+^, C_15_H_22_O_4_; ^1^H (500 MHz) and ^13^C (125 MHz) NMR data, see Table 1.

### Computational details

Conformational search was done using Molecular Merck force field (MMFF) embedded in Spartan'14 software (Wavefunction Inc., Irvine, CA, USA). Density functional theory (DFT) and time-dependent density functional theory (TDDFT) calculations were performed with Gaussian09 RevD.01 [42]. Double-hybrid (DH) DFT calculations were conducted with ORCA 5.0.4 program package using RIJCOSX approximation, tight SCF criteria [43]. For conformational analysis, conformers of compounds **1**, **2** and **3** each within an 8 kcal/mol energy window from MMFF conformational search were subjected to geometry optimizations followed by frequency calculations using DFT method at the B3LYP [44, 45] -GD3BJ [46, 47] /6-31G(d) level of theory. To simulate the ^13^C NMR shifts of **3**, the optimized low-energy conformers (relative electronic energies < 4.0 kcal/mol) were subjected to NMR calculations using the gauge including atomic orbitals (GIAO) [15, 17, 18] method at the mPW1PW91/6–311 + G (d, p) level [16] of theory with the solvent model PCM for chloroform. The unscaled chemical shifts (*δ*_u_) were computed using TMS as reference standard according to *δ*_u_ = σ_0_ − σ^x^ (where σ^x^ is the Boltzmann averaged shielding tensor and σ_0_ is the shielding tensor of TMS computed at the same level employed for σ^x^). The Boltzmann averaging was done using the relative energies obtained from the single-point NMR calculations [19, 20] The goodness of fit test between the simulated ^13^C NMR data of the four stereoisomers and the experimental shifts of **3** were evaluated by the improved DP4 probability (DP4 +) [19, 20]. For calculations of the ECD spectra, the optimized geometries were subjected to single point calculations using the DH-DFT method at the (PWPB95 [48]-GD3BJ/def2-QZVPP [49] level of theory with the SMD [50] solvent model for MeOH to obtain more accurate electronic energies. The TDDFT calculations were carried out using M06-2X [51], PBE1PBE (PBE0) [52], M11-L [53], and revTPSS [54] functionals in combination with the TZVP [51] basis set and the PCM solvation model for MeOH. The number of excited states was 30 for all conformers. The results were visualized and exported using the SpecDis program [55]. The calculated ECD spectra was generated as a sum of Gaussian curve using rotatory strengths computed in the dipole-velocity gauge from ECD data of the individual conformers. Boltzmann distributions of the conformers in equilibrium population were estimated from the relative Gibbs free energies (Δ*G*) at 298.15K.

### Antioxidant activity

Antioxidant activity was evaluated by in vitro DPPH radical scavenging assay, ABTS radical cation scavenging assay, and cellular reactive oxygen species (ROS) scavenging assay following our lab previous methods [56, 57].

### *α*-Glucosidase activity

The *α*-glucosidase inhibitory activity of compounds **1–19** was assessed based on a previously reported method with slight modifications [58]. In a 96-well microtiter plate, 70 *µ*L of phosphate buffer, 10 *µ*L of *α*-glucosidase enzyme solution (0.5 U/mL), and 10 *µ*L of the test compound at varying concentrations (50, 25, 12.5, 6.25, and 3.12 *μ*M) were added to each well. Corosolic acid (10 *µ*L) was used as a positive control. For the negative control, the test compound was replaced with phosphate buffer, while the blank control was prepared by omitting the enzyme and substituting it with buffer. The reaction mixtures were incubated at 37 °C for 10 min, followed by the addition of 10 *µ*L of PNPG solution per well. After a further 20 min incubation at 37 °C, the reaction was terminated by adding 100 *µ*L of Na_2_CO_3_ solution. The absorbance was measured at 405 nm using a microplate reader. All experiments were conducted in triplicate, and results are expressed as mean values ± standard deviation (SD).

$${\text{Inhibition }}\left( \% \right) \, = \left[ {1 - \frac{{(OD_{sample} - OD_{blank} )}}{{\left( {OD_{negative} - OD_{blank} } \right)}}} \right] \times 100 \%$$

### Antibacterial activity

The antibacterial activity of compounds **1**–**19** was evaluated against *Staphylococcus aureus* (MSSA, CMCC 26003), methicillin-resistant *Staphylococcus aureus* (MRSA, JCSC 3063), and *Escherichia coli* (EC, ATCC 8739) using a resazurin-based microdilution assay. Bacterial suspensions were prepared by adding 30 *µ*L of the inoculum to 5 mL of Brain Heart Infusion (BHI) medium followed by incubation at 37 °C in a shaking incubator set at 200 rpm for 12 h. The bacterial suspension was then diluted to an optical density (OD) of 0.07 ± 0.002, corresponding to 1.25 × 10^6^ CFU/mL. A resazurin stock solution (1 mg/mL) was prepared in sterile water and diluted to 100 *µ*g/mL for use. Stock solutions of compounds were prepared in biological-grade DMSO at 1 mM, and used as test samples. The assay was performed on 96-well plates. First, 90 *µ*L of a resazurin-bacterial mixed solution (in a 3:2 volume ratio) and 10 *µ*L of the test samples were added to the first row (Row A) and thoroughly mixed using a pipette. Then, 50 *µ*L of the resazurin-bacterial solution was added to rows B–H. Next, 50 *µ*L mixture from Row A was transferred to Row B and mixed well; this process was repeated for Rows C–H. Two-fold serial dilutions were performed across the rows. The plate was incubated at 37°C for 6–8 h. The MIC was determined by the lowest concentration at which the bacterial solution changed from purple to pink, indicating inhibition of growth. Each test was performed in triplicate. All bacterial strains were obtained from the Guangdong Institute of Microbiology (Guangzhou, China). Vancomycin and kanamycin were used as positive controls for MSSA and MRSA, and polymyxin for EC.

### Anti-inflammatory activity

The anti-inflammatory potential of compounds **1**–**3** was evaluated by measuring their ability to inhibit nitric oxide (NO) production in lipopolysaccharide (LPS)-stimulated RAW 264.7 macrophages, following our previously established protocols [57].

## Conclusion

In conclusion, the phytochemical exploration of *H. rhamnoides* roots yielded three new 14-noreudesmane-type sesquiterpenoids and a suite of known compounds. The main constituents were oleanane-type triterpenoids. While the newly identified sesquiterpenoids exhibited limited biological activity, several triterpenoids demonstrated promising *α*-glucosidase inhibitory, and antibacterial properties. Hippophamide (**17**) showed significant antioxidant activity with DPPH and ABTS radicals scavenging potential. These results enriched the chemical profile of *H. rhamnoides* roots.

## Supplementary Information

Supplementary material 1

## Data Availability

The experimental data supporting this work are accessible within the article and its Additional file 1.
