# Supplementary material for: Three new 14-noreudesmane-type sesquiterpenoids from the roots of Hippophae rhamnoides
Source: Nat Prod Bioprospect. 2026 Feb 4;16(1):30. doi: 10.1007/s13659-025-00581-0 (PMC12868457; doi:10.1007/s13659-025-00581-0)
Supplement: Supplementary file 1 — Supplementary material 1 [file 13659_2025_581_MOESM1_ESM.docx]

**Natural Products and Bioprospecting**

**Three New 14-Noreudesmane-Type Sesquiterpenoids from the Roots of *Hippophae rhamnoides***

**Appendix A, Supplementary data**

**Fatima Abdurrahman Galadanchi^a,b,c^, Polina Lopukhina^a,b,c^, Sisi Bai^a,b,c^, Guohao Dong^a,b,c^, Zhongyu Zhou^a,b,c^ *, Haihui Xie^a,b,c^, Xiaoyi Wei^a,b,c^**

*^a^ Guangdong Provincial Key Laboratory of Applied Botany & Key Laboratory of National Forestry and Grassland Administration on Plant Conservation and Utilization in Southern China, South China Botanical Garden, Chinese Academy of Sciences, Guangzhou 510650, China*

*^b^ South China National Botanical Garden, Guangzhou 510650, China*

*^c^ University of Chinese Academy of Sciences, Beijing 100049, China*

Corresponding e-mail: [zhouzhongyu@scbg.ac.cn](mailto:zhouzhongyu@scbg.ac.cn)

**Fig. S1** *Hippophae rhamnoides* roots

**Fig. S2** The structures of known compounds **(4**–**19)** isolated from the roots of *H. rhamnoides*

**Fig. S3** Flow chart of the extraction and isolation procedure of compounds **1**–**19**

**Fig. S4** ^1^H NMR spectrum of compound **1**

**Fig. S5** ^13^C NMR spectrum of compound **1**

**Fig. S6** HSQC spectrum of compound **1**

**Fig. S7** HMBC spectrum of compound **1**

**Fig. S8 ^1^**H-^1^H COSY spectrum of compound **1**

**Fig. S9** ^1^H NMR spectrum of compound **2**

**Fig. S10** ^13^C NMR spectrum of compound **2**

**Fig. S11** HSQC spectrum of compound **2**

**Fig. S12** HMBC spectrum of compound **2**

**Fig. S13 ^1^**H-^1^H COSY spectrum of compound **2**

**Fig. S14** ^1^H NMR spectrum of compound **3**

**Fig. S15** ^13^C NMR spectrum of compound **3**

**Fig. S16** HSQC spectrum of compound **3**

**Fig. S17** HMBC spectrum of compound **3**

**Fig. S18 ^1^**H-^1^H COSY spectrum of compound **3**

**Fig. S19** HR-EI-MS spectrum of compound **1**

**Fig. S20** EI-MS spectrum of compound **2**

**Fig. S21** HR-EI-MS spectrum of compound **2**

**Fig. S22** ESI-MS (positive) spectrum of compound **3**

**Fig. S23** HR-ESI-MS (positive) spectrum of compound **3**

**Fig. S24** UV spectrum of compound **1**

**Fig. S25** UV spectrum of compound **2**

**Fig. S26** UV spectrum of compound **3**

**Fig. S27** IR spectrum of compound **1**

**Fig. S28** IR spectrum of compound **2**

**Fig. S29** IR spectrum of compound **3**


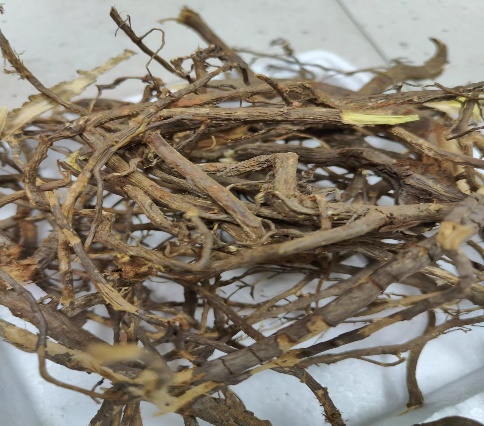


**Fig. S1** *Hippophae rhamnoides* roots


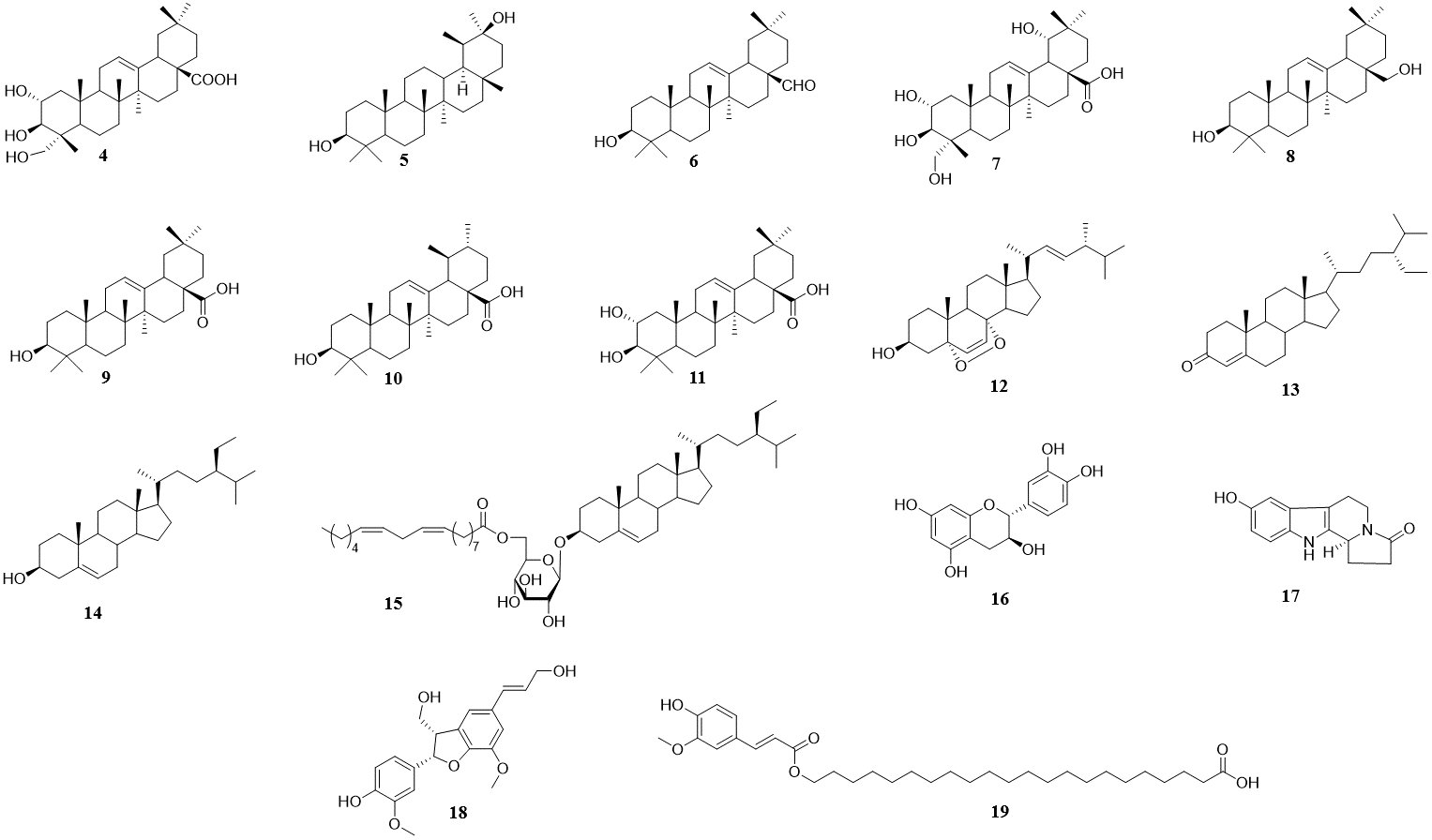


**Fig. S2** The structures of known compounds (**4**–**19**) isolated from the roots of *H.rhamnoides*. They are arjunolic acid (**4**), 18H*α*,3*β*,20*β*-ursanediol (**5**), 3*β*-hydroxyolean-12-en-28-al (**6**), 2*α*,3*β*,19*α*,23-tetrahydroxyolean-12-en-28-oic acid (**7**), erythrodiol (**8**), oleanolic acid (**9**), ursolic acid (**10**), maslinic acid (**11**), ergosterol endoperoxide (**12**), stigmast-4-en-3-one (**13**), *β*-sitosterol (**14**), daucosterol-6'-linoleate (**15),** (+)-catechin (**16**), hippophamide (**17**), dehydrodiconiferyl alcohol (**18**), 22-*O*-(4-hydroxy-3-methoxy-cinnamy1) docosanoic acid (**19**)


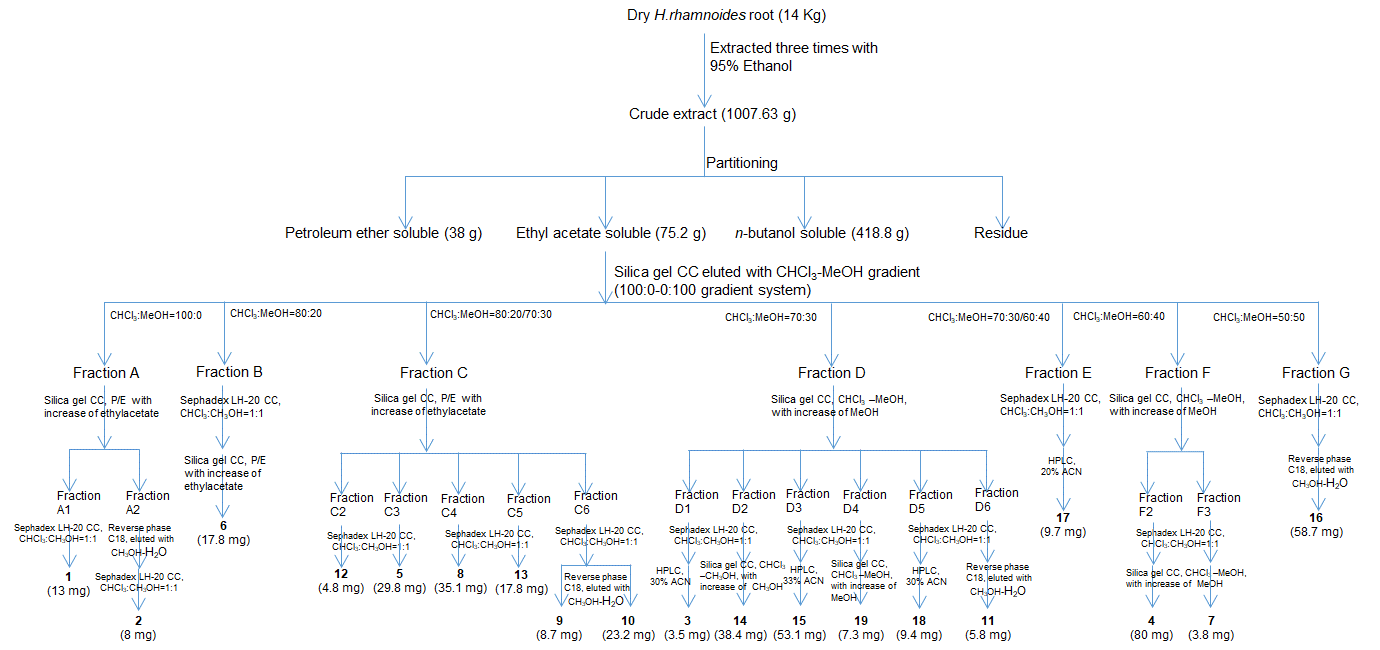


**Fig. S3** Flow chart of the extraction and isolation procedure of compounds **1**–**19**

CC = Column Chromatography, ACN = Acetonitrile


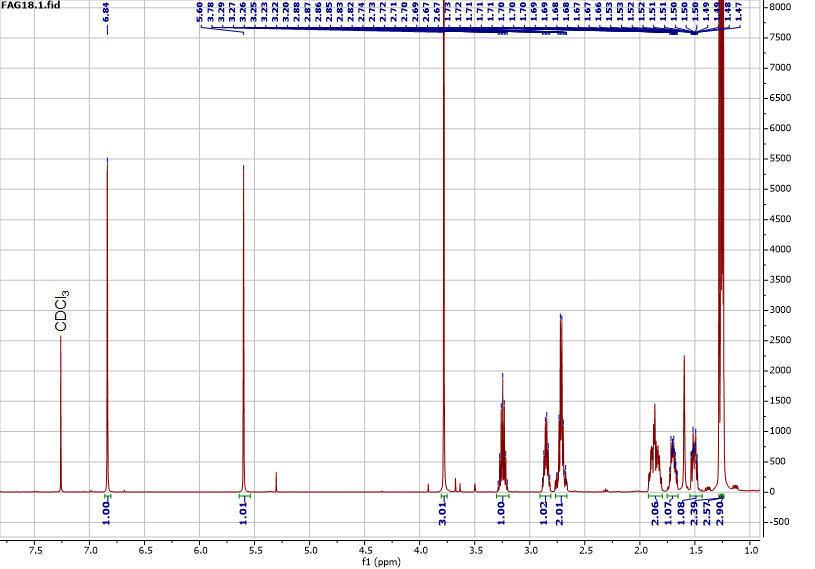


**Fig. S4** ^1^H NMR spectrum of compound **1**

**Fig. S5** ^13^C NMR spectrum of compound **1**

**Fig. S6** HSQC spectrum of compound **1**

**Fig. S7** HMBC spectrum of compound **1**

**Fig. S8 ^1^**H-^1^H COSY spectrum of compound **1**


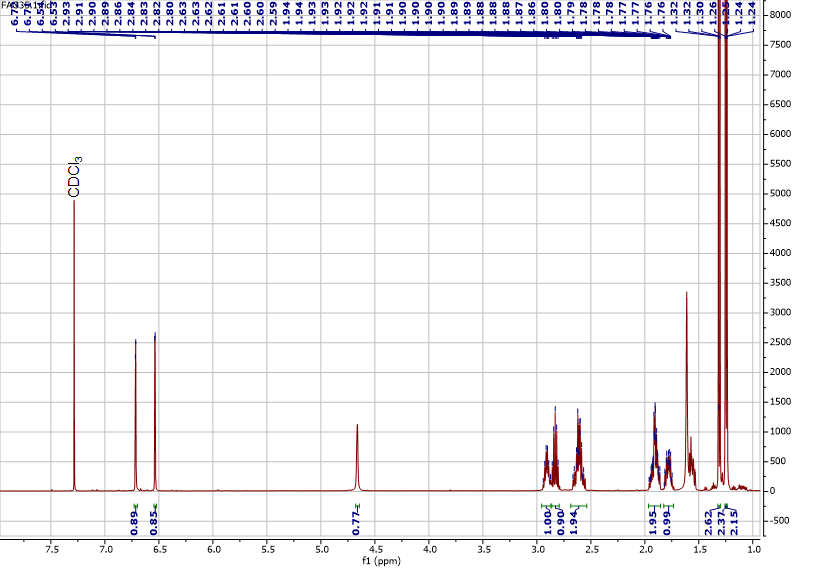


**Fig. S9** ^1^H NMR spectrum of compound **2**

**Fig. S10** ^13^C NMR spectrum of compound **2**

**Fig. S11** HSQC spectrum of compound **2**

**Fig. S12** HMBC spectrum of compound **2**

**Fig. S13 ^1^**H-^1^H COSY spectrum of compound **2**


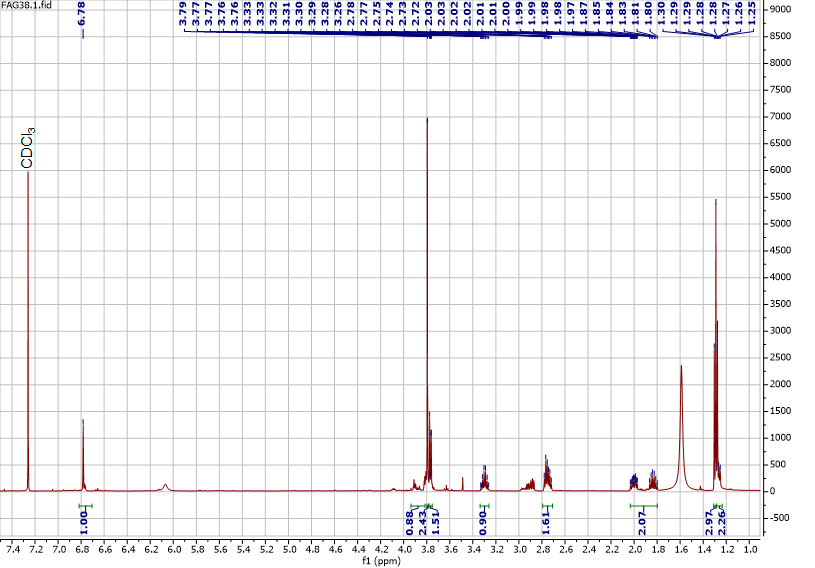


**Fig. S14** ^1^H NMR spectrum of compound **3**

**Fig. S15** ^13^C NMR spectrum of compound **3**

**Fig. S16** HSQC spectrum of compound **3**

**Fig. S17** HMBC spectrum of compound **3**

**Fig. S18 ^1^**H-^1^H COSY spectrum of compound **3**

**
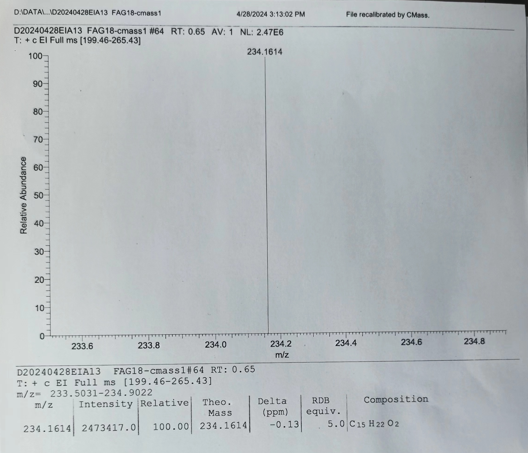
**

**Fig. S19** HR-EI-MS spectrum of compound **1**


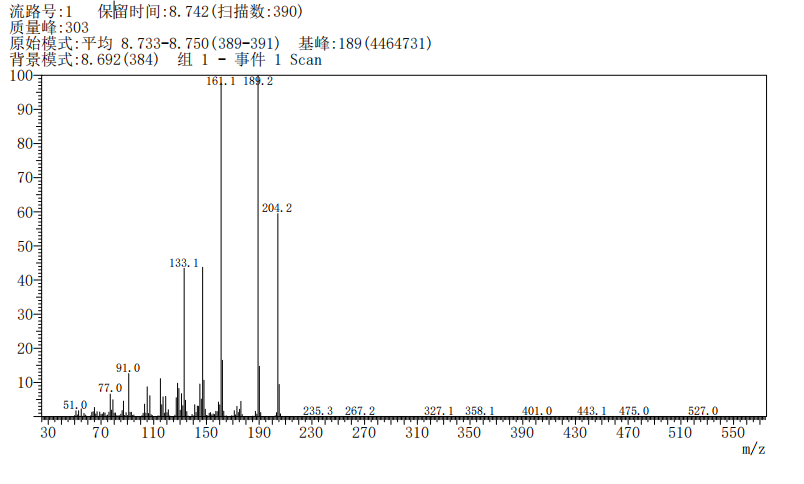


**Fig. S20** EI-MS spectrum of compound **2**


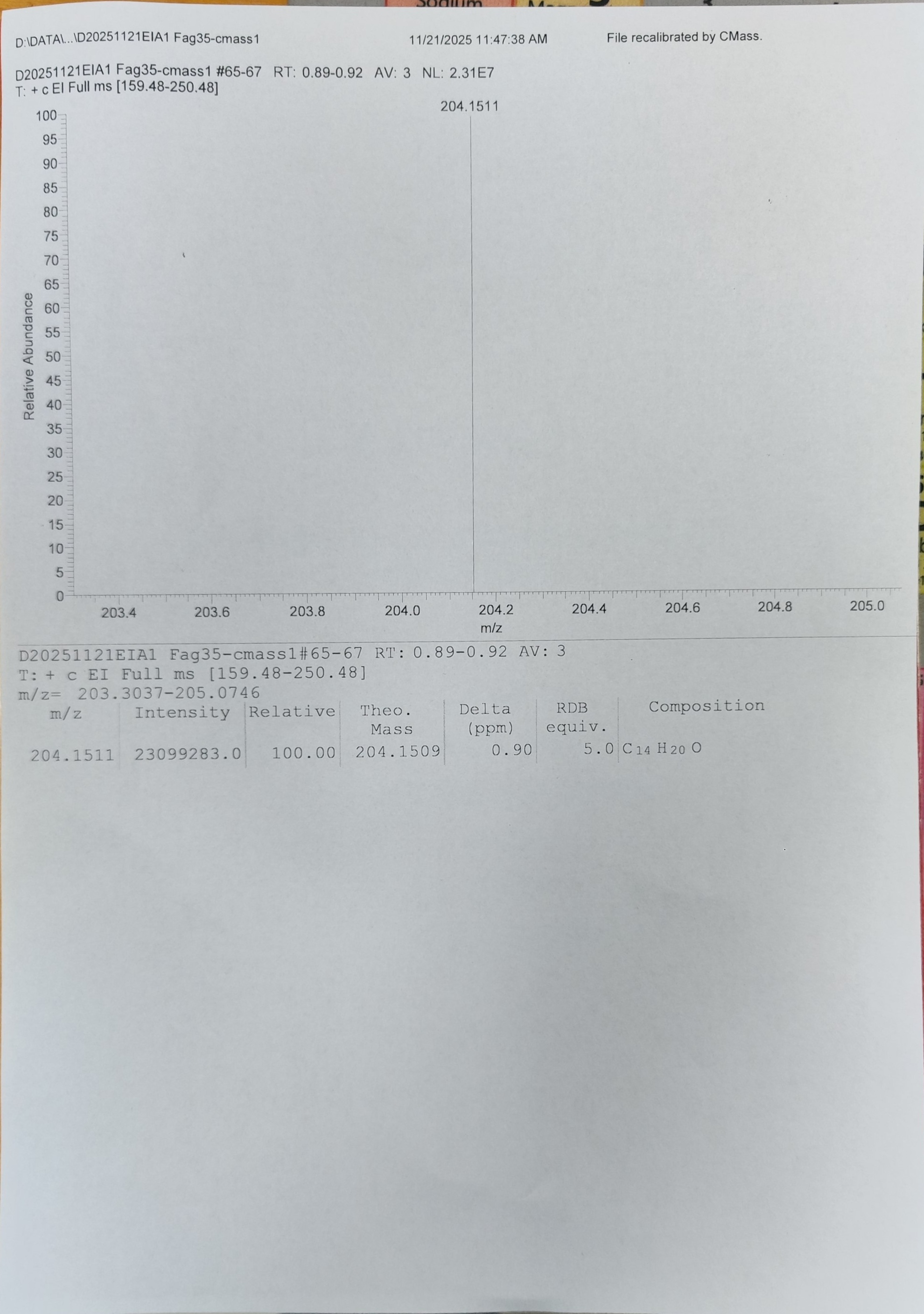


**Fig. S21** HR-EI-MS spectrum of compound **2**

**Fig. S22** ESI-MS (positive) spectrum of compound **3**


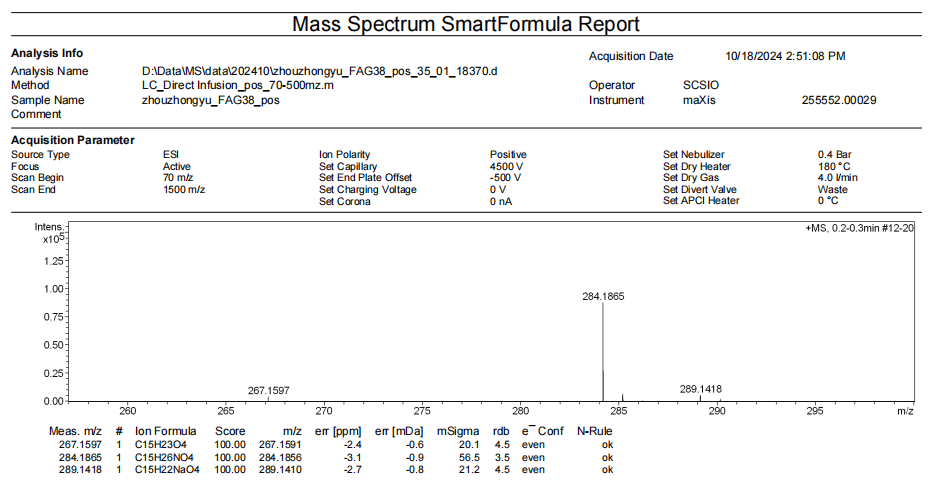


**Fig. S23** HR-ESI-MS (positive) spectrum of compound **3**


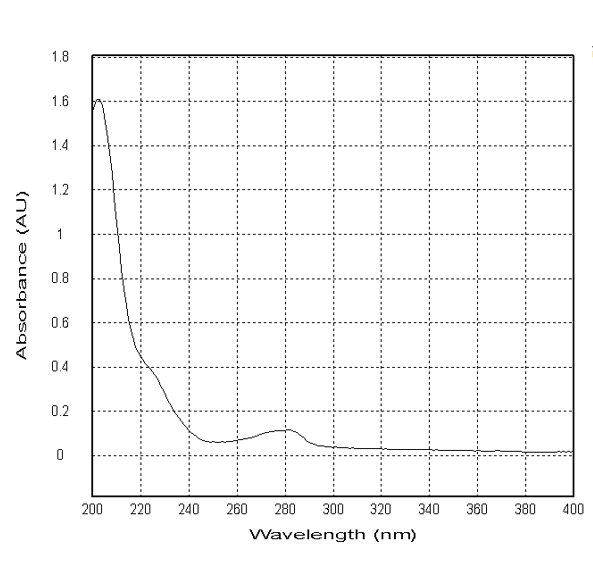


**Fig. S24** UV spectrum of compound **1**


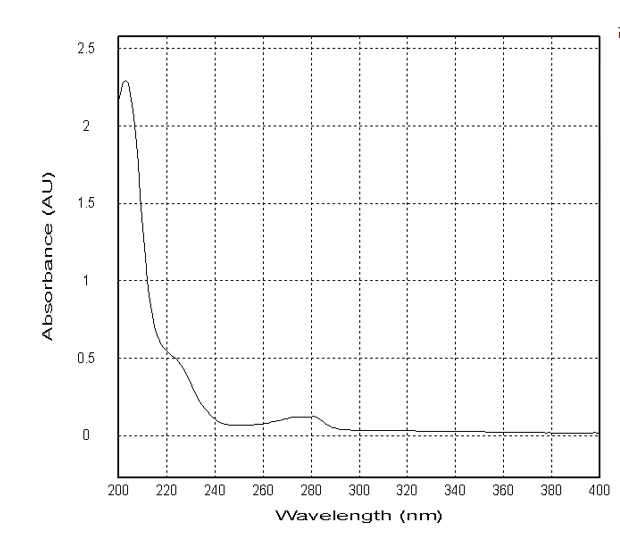


**Fig. S25** UV spectrum of compound **2**


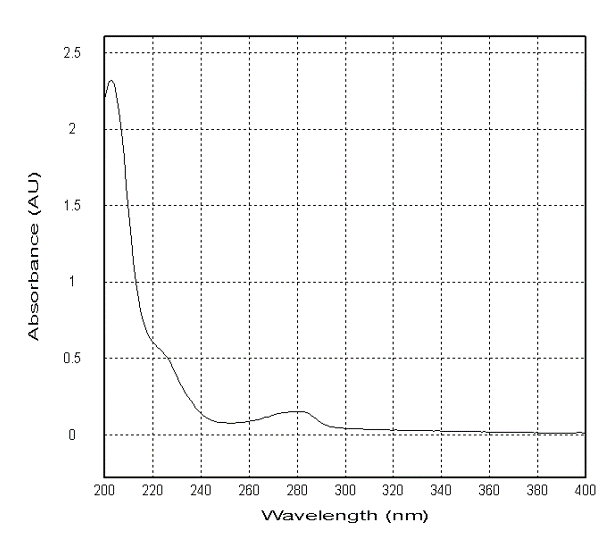


**Fig. S26** UV spectrum of compound **3**

**Fig. S27** IR spectrum of compound **1**

**Fig. S28** IR spectrum of compound **2**

**Fig. S29** IR spectrum of compound **3**
